# A Synthetic Method to Access Symmetric and Non-Symmetric 2-(*N*,*N'*-disubstituted)guanidinebenzothiazoles

**DOI:** 10.3390/molecules170910178

**Published:** 2012-08-24

**Authors:** Alejandro Cruz, Itzia I. Padilla-Martínez, Efrén V. García-Báez

**Affiliations:** Departamento de Ciencias Básicas de la Unidad Profesional Interdisciplinaria de Biotecnología del IPN, Av. Acueducto s/n, Barrio la Laguna Ticomán, México, D.F., 07340, Mexico

**Keywords:** 2-aminobenzothiazole, dithiomethylcarboimidate, isothiourea, benzothiazole, guanidine

## Abstract

Symmetric and non-symmetric 2-(*N*-H, *N*-methyl, *N*-ethylenyl and *N*-aryl)guanidinebenzothiazoles were synthesized from the reaction of ammonia, methylamine, pyrrolidine and aniline with dimethyl benzo[*d*]thiazol-2-yl-carbono-dithioimidate (**5**) as intermediate. The products were characterized by ^1^H-, ^13^C-NMR spectroscopy and three of them by X-ray diffraction analysis. H*N*-phenyl protons formed intramolecular hydrogen bonds that assist the stereochemistry of the second substituent, whereas the H*N*-alkyl protons were involved in intermolecular hydrogen bonding.

## 1. Introduction

The guanidine group has attracted considerable attention since it is found in a wide array of natural and synthetic biologically active compounds [1,2,3]. The guanidine groups are categorized as organosuperbases whose basicity is magnified because of the resonance stabilization of the corresponding conjugated acids. These molecules are basic enough (pKa of their conjugated acids is around 12.5) to form intermolecular contacts mediated by H-bonding interactions [4]. Its positive charge, resulting from protonation in a wide range of pH values, plays an important role in forming specific intermolecular interactions, comprising key-steps of many biological reactions including enzyme-mediated processes and interaction of hormones with their receptors [5]. The guanidinium moiety interacts with functional groups present in enzymes or receptors on the basis of hydrogen bonds and electrostatic interactions to form intermolecular associations. Thus, they are useful pharmacophores in medicinal chemistry [6]. Moreover, synthetic guanidines have found wide applications in the engineering of advanced synthetic molecular recognition devices, sensors, organic materials and phase-transfer catalysts [7,8]. Recently, it has been demonstrated that by introduction of chirality in one of the guanidinyl nitrogen atoms [9,10,11], the resulting chiral guanidines were effective in catalytic [12,13,14,15] and stoichiometric asymmetric synthesis [16,17]. Due to this, continued interest has been shown in the transformation of amines into the corresponding guanidines, because the guanidine group, instead of an existing amino group, can significantly increase the potency and/or selectivity of biologically active compounds [18,19,20].

**Figure 1:**
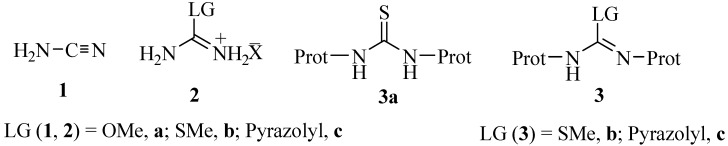
Electrophilic amidine species used to generate guanidines from amines, LG = leaving group.

Typically, the synthesis of guanidine-containing compounds involves the treatment of an amine with an electrophilic amidine species. The most commonly used reagents include cyanamide (**1**) [21], *O*-methylisourea hydrogen sulfate (**2a**) [22], *S*-methyl isothiouronium salts (**2b**) [23,24,25], pyrazole-1-carboxamidine (**2c**) [26], *N*-protected thiourea (**3a**) [27,28,29], (*S*-methyl or -aryl)isothiourea (**3b**) [30,31,32,33] or pyrazole-1-carboxamidine (**3c**) derivatives [34,35] (Figure 1). To increase yields, new reagents with electron-withdrawing substituents have been developed in the recent years [36,37].

The synthesis of guanidines from thioureas and isothioureas are the most commons strategies used for the construction of the guanidine functionality. In the case of thioureas, they are activated through the reaction with DIC, EDCI, Hg^2+^ (most popular but toxic), 2-chloro-1-methylpyridinium iodide, 2,4-dinitrofluorobenzene, *etc.* Recently, a bismuth catalyst has been found to afford high yields (70%–97%) [38]. The attachment of thiourea groups to a solid phase has been used as precursor of guanidinium groups [39]. Recently, it has been demonstrated that guanylilation of an amine from a thiourea, involves the attack of the amine on what is generally accepted to be a carbodiimide intermediate [40,41,42,43,44,45,46].

Diphenylcarbodiimide has been used for the synthesis of diphenylguanidinobenzothiazole [47] and dicyandiamide for the synthesis of 2-guanidinobenzothiazole [48]. However, to the best of our knowledge, there is no reports about non-symmetrical guanidines derived from 2-aminobenzothiazole.

**Scheme 1:**
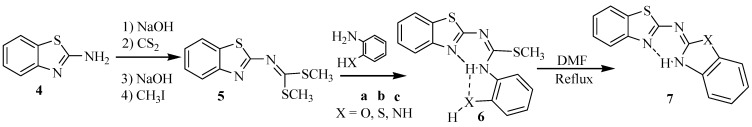
NH-bisbenzazoles from dimethyl benzo[*d*]thiazol-2-ylcarbonodithioimidate (**5**).

**Scheme 2:**
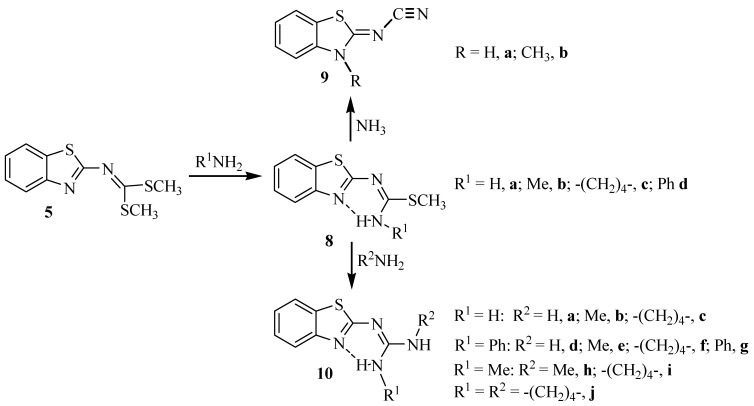
*S*-methylisothioureas **8**, cyanamides **9** and guanidines **10** derived from 2-aminobenzothiazole **4**, through dimethyl benzo[*d*]thiazol-2-ylcarbonodithioimidate **5**.

We have reported [49] a detailed study and characterization of the intermediates involved in the synthesis of dimethyl benzo[*d*]thiazol-2-ylcarbonodithioimidate (**5**), from the reaction of 2-aminobenzothiazole (**4**) with carbon disulfide in basic media, using DMF as solvent [50]. Two molecules of HSMe are displaced when **5** reacts with *o*-XH substituted anilines in refluxing DMF to get NH-bisbenzazoles **7** [51,52,53] (Scheme 1). We found that the reaction proceeds through the intermediacy of isothiourea derivatives **6a**–**c** when *o*-XH anilines are refluxed 16 h in ethanol. Under these conditions, isothiourea **6c** could be isolated and the reaction was extended to *m*- and *p*-phenylenediamines [54]. In the same work, we reported the synthesis of S-methyl-*N*-alkylbenzothiazolyl-isothioureas **8a**–**d** when ammonia, methylamine, pyrrolidine, aniline and 1,4-piperazine were used (Scheme 2).

In continuation of this work, we were interested in extending the routes of synthesis of guanidine compounds, in this sense, we generalized this reaction to prepare symmetrical **10g**,**h**,**j** and nonsymmetrical **10b**–**f**,**i** guanidine derivatives obtained from 2-aminobenzothiazole (**4**, Scheme 2). Herein we report the preparation and ^1^H and ^13^C-NMR structural study of a series of guanidines **10b**–**j**. The isolation of *S*-methylisothiourea intermediates **8** is important since their remaining reactive *S*-methyl group was subsequently substituted by amines to form the guanidine group.

## 2. Results and Discussion

When dithiocarboimidate **5** reacts with one molar equivalent of ammonia, alkylamine or aniline in ethanol, one molar equivalent of thiomethanol was evolved to afford the corresponding S-methylbenzothiazolyl-isothiourea **8a**–**d** as isolable intermediates (Scheme 2). Ammonia required 72 h and alkylamine 48 h on stirring at room temperature, to be completed, whereas aniline required 24 h in refluxing ethanol.

**Figure 2:**
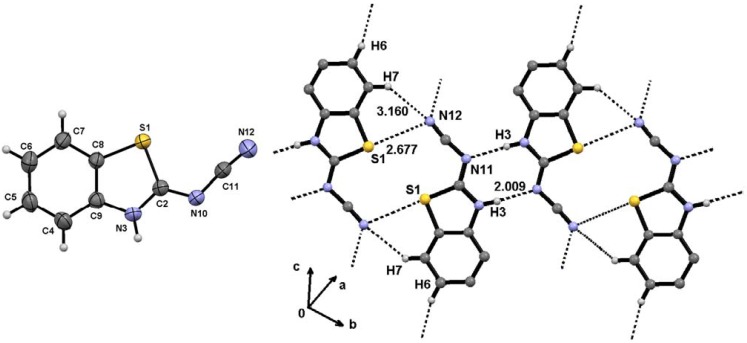
Molecular structure of 2-cyanamidabenzothiazole **9a** (**left**) and hydrogen bonding scheme (**right**). Selected bond lengths (Å) and angles (°): S(1)-C(2) = 1.744(2), N(3)-C(2) = 1.338(3), N(10)-C(2) = 1.311(3), N(10)-C(11) = 1.337(3), N(12)-C(11) = 1.155(3), C(2)-N(10)-C(11) = 118.90(19), S(1)-C(2)-N(3) = 112.59(16), S(1)-C(2)-N(10) = 126.44(18), N(3)-C(2)-N(10) = 121.0(2), S(1)-C(8)-C(9) = 111.40(16), N(3)-C(9)-C(8) = 111.69(19), N(10)-C(11)-N(12) = 174.3(3), C(8)-S(1)-C(2)-N(3) = 0.40(17), C(8)-S(1)-C(2)-N(10) = 179.7(2), S1-C2-N10-C11 = −1.0°(3), C(11)-N(10)-C(2)-N(3) = 178.3(2).

The reaction of isothiourea intermediates **8a**–**d** with a second equivalent of several amines (ammonia, methylamine, aniline and pyrrolidine) was then carried out. When the reaction of isothiourea intermediate **8a** was performed with excess ammonia in refluxing ethanol, 2-cyanamido-benzothiazole **9a** crystallized as the only product in a 62% yield. The X-ray diffraction structure of **9a** is shown in Figure 2, along with a summary of representative distances and angles. The N12-C11 bond length is the shortest N–C bond, its value of 1.155(3) Å is in the characteristic range of a triple bond [55], confirming the presence of the cyano group. In addition, N10-C11 [1.337(3) Å] and N3-C2 [1.338(3) Å] are in agreement with a single bond whereas the shorter N10-C2 [1.311(3) Å] bond length is more appropriate for a double bond character. On the other hand, the X-ray diffraction structure of **9a** shows that the mobile hydrogen atom prefers to stay on the benzothiazole nitrogen because of two intermolecular interactions, N3-H3∙∙∙N10, stabilize the molecule as a dimer [H3∙∙∙N10 = 2.01 Å, N3∙∙∙N10 = 2.866(2) Å, N3-H3∙∙∙N10 = 174°; symmetry code: –x, −y, 1−z]. In this arrangement, the nitrile group is *cis* positioned to the sulfur atom, thus the electronic conjugation is extended from the benzothiazole system to the nitrile group. The torsion angles S1-C2-N10-C11 [−1.0°(3)] and C11-N10-C2-N3 [178.3°(2)] are in agreement with a planar molecule. The nitrile is a polarized functional group whose interaction with sulfur atom, as Lewis acid, is favored by *cis* configuration [N12∙∙∙S1 = 3.160(3) Å] and C7-H7∙∙∙N12 interaction [H7∙∙∙N12 = 2.677 Å, C7-N12 = 3.343(4) Å, C7-H7∙∙∙N12 = 138°; symmetry code: 1−x, 1−y, 1−z].

**Scheme 3:**
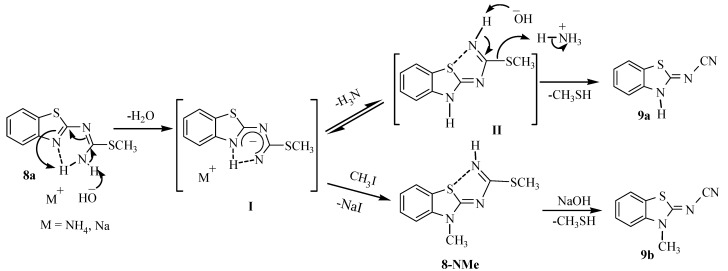
Thioamidines **II** and **8-NMe** as intermediates in the formation of 2-cyanoimino-benzothiazoles **9a** and **9b**.

Compound **9a** is formed when *S*-methylisothiourea **8a** suffers HSMe group elimination promoted by the basic media (Scheme 3). The proposed mechanistic pathway involves the participation of NH_4_OH to remove one NH hydrogen atom from **8a** to *in situ* form the ammonium intermediate **I**, which is stabilized as the tautomer intermediate **II**. A second molecule of NH_4_OH promotes the elimination of the HSMe group to generate the nitrile **9a**. The intermediate **II** was transformed to **8-NMe**, (*δ* = 3.80 NMe, 2.66 SMe) by reaction with one molar equivalent of NaOH and CH_3_I, which is readily transformed into 2-cyanamide-*N*-methylbenzothiazole compound **9b** by a second molar equivalent of NaOH (Scheme 3).

Compound **9a** was characterized in solution by NMR. The nitrile carbon atom appears as a small signal at 117.7 ppm, very similar to the observed value of 118.7 ppm in benzonitrile [56]. The mass spectrometry [M^+^ = 175 *m/z* (100%)] and elemental analysis, are in agreement with the proposed structure. Under the same conditions already described for **8a**, isothioureas **8b** and **8c** failed to react with an excess of ammonia to give nonsymmetrical guanidines **10b** and **10c** respectively, and the starting materials were recovered, however, the reaction of isothiourea **8d** with an excess of ammonia, affords the nonsymmetrical guanidine **10d** as the only product. In this case, the acidic aniline hydrogen is intramolecularly engaged with the benzothiazole nitrogen atom. This hydrogen bonding interaction is strong enough to polarize the imine carbon and favors the substitution of the SMe group by ammonia.

On the basis of this result, the reaction of isothiourea **8a** with methylamine and pyrrolidine were carried out to get nonsymmetrical guanidines **10b** and **10c**, after refluxing in ethanol for 4 and 2 days, respectively. The reaction of **8a** with aniline failed to give the corresponding guanidine compound **10d**, even after 4 days in refluxing ethanol. Under these conditions, aniline is not nucleophilic enough to add to the carbodiimide intermediate **II**. The reaction of isothiourea **8b** or **8c** with one molar equivalent of methylamine or pyrrolidine in refluxing ethanol for 8 h afforded the corresponding guanidines **10h**,**i** or guanidines **10i**,**j**, respectively. Symmetric guanidines **10h**,**j** can also be obtained when dithiocarboimidate **5** is reacted with two molar equivalents of the corresponding amine in refluxing ethanol for 8 h.

The reaction of isothiourea **8d** with one molar equivalent of methylamine, pyrrolidine and aniline was tested. After 3 days in refluxing ethanol, the corresponding nonsymmetrical guanidines **10e** and **10f**, were obtained. The reaction with aniline required harsh conditions: refluxing DMF or solventless heating. In the ^1^H-NMR spectrum of **10g**, only aromatic protons and a broad signal at 12.4 ppm, assigned to the N-H protons, were observed. Moreover, sixteen signals in the ^13^C-NMR spectrum are indicative of the presence of the 2-*N,N*-diphenyl guanidinebenzothiazole **10g** (Table 1 and Table 2). The mass spectrometry data [M^+^ = 344 *m/z* (19%)] is in agreement with the proposed structure.

**Table 1:** ^1^H-NMR chemical shifts of compounds **9a**–**b**, **10b**–**j**. 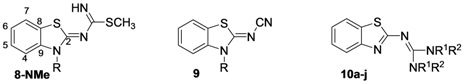

Comp.	H4	H5	H6	H7	NH	NCH_3_, SCH_3_	NPh	N(CH_2_CH_2_)_2_
**8-NMe** **^a^**	8.00	7.76	7.61	7.46	9.8	3.80, 2.66		
**9a** **^a^**	7.75	7.27	7.24	7.39				
**9b** **^b^**	7.58	7.46	7.31	7.24		3.62		
**10b** **^a^**	7.62	7.21	7.03	7.42	7.7	2.75		
**10c** **^a^**	7.63	7.21	7.04	7.44	8.2			3.4, 1.8
**10d** **^b^**	7.61	7.30	7.14	7.59			7.4–7.3	
**10e** **^b^**	7.69	7.30	7.17	7.63	11.2	2.97	7.5–7.3	
**10f** **^b^**	7.66	7.32	7.18	7.63	11.6		7.3–7.1	3.4, 1.8
**10g** **^a^**	7.73			7.31	11.9		7.4–7.3	
**10h** **^b^**	7.61	7.24	7.10	7.55	9.6	2.92		
**10i** **^b^**	7.60	7.26	7.08	7.57	8.9	3.53		3.0, 1.9
**10j** **^b^**	7.51	7.20	7.00	7.49				3.4, 1.8

^a^ DMSO-*d*_6_; ^b^ CDCl_3__。_

**Table 2:** ^13^C-NMR chemical shift of compounds **9a**–**b**, **10b**–**j**, in DMSO-*d*_6_.

Comp.	C2	C4	C5	C6	C7	C8	C9	C11	NCH_3_, SCH_3_	Ph
**8-NMe** ^a^	166.0	114.6	124.1	124.6	128.7	126.2	138.5	175.1	33.6, 15.7	
**9a** ^a^	174.0	114.0	123.7	124.4	128.2	125.0	139.4	117.7		
**9b** ^b^	171.5	112.2	122.9	124.8	127.9	123.3	139.4	116.9	31.3	
**10b** ^a^	158.4	125.9	122.3	121.4	118.9	130.9	152.5	174.3	28.3	
**10c** ^a^	155.4	125.9	122.2	121.4	119.0	130.9	152.6	174.4		
**10d** ^b^	156.0	125.7	122.7	122.1	119.6	131.3	151.8	173.7		136.8, 130.2, 125.7, 127.0
**10e** ^b^	154.6	125.6	122.5	121.2	119.5	131.7	151.9	174.5	28.6	137.0, 130.2, 126.9, 126.0
**10f** ^b^	154.4	125.6	122.4	121.1	119.6	132.0	151.9	173.8		139.9, 129.5, 125.6, 123.3
**10g** ^a^	151.5	125.8	121.5	121.3	119.9	132.0	151.0	173.6		137.3, 129.8, 123.6, 123.0
**10h** ^b^	157.2	125.5	122.2	121.0	119.1	131.6	152.2	174.7	28.2	
**10i** ^b^	159.2	125.4	122.0	121.0	119.1	132.1	152.2	173.6	31.5	
**10j** ^b^	158.0	125.2	121.2	121.8	119.1	133.4	153.3	171.3		

N(CH_2_CH_2_): 46.9, 25.5 (**10c**); 49.3, 25.6 (**10f**); 49.2, 25.7 (**10i**); 49.6, 25.6 (**10j**). ^a^ DMSO-*d*_6_; ^b^ CDCl_3__。_

Comparison of ^13^C-NMR chemical shifts of guanidine compounds **10** to those of dimethyl benzo[*d*]thiazol-2-ylcarbonodithioimidate **5**, shows that C2 and C11 are shifted to low frequencies by approximately 10 and 2 ppm, respectively (Table 2). This effect is explained by the extended conjugation of N12 and N13 electron pairs to the benzothiazole ring, increasing the electronic protection of C2 atom. This effect also shifts C7 and C8 to low frequencies by 6 and 3 ppm, respectively. In contrast, C2 is shifted by 6.5 ppm to higher frequencies in 2-aminonitrilebenzothiazole **9a**.

**Figure 3:**
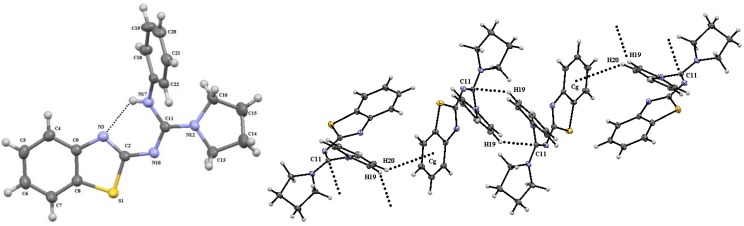
Molecular structure of guanidine **10f** (**left**) and intermolecular interactions (**right**). Selected bond lengths (Å) and angles (°): S(1)-C(2) = 1.774(2), N(3)-C(2) = 1.310(3), N(10)-C(2) = 1.354(3), N(10)-C(11) = 1.332(3), N(12)-C(11) = 1.343(3), N(17-C(11) = 1.360 (3), C(2)-N(10)-C(11) = 121.06(17), S(1)-C(2)-N(3) = 114.34(15), S(1)-C(2)-N(10) = 114.72(14), N(3)-C(2)-N(10) = 130.94(18), S(1)-C(8)-C(9) = 109.07(15), N(3)-C(9)-C(8) = 115.67(18), N(10)-C(11)-N(12) = 116.97(17), C(8)-S(1)-C(2)-N(3) = −0.82(16), C(8)-S(1)-C(2)-N(10) = 179.46(15), S1-C2-N10-C11 = −172.69°(15), C(11)-N(10)-C(2)-N(3) = 7.7(3), N(12)-C(11)-N(10)-C(2) = −179.62(17), N(17)-C(11)-N(10)-C(2) = −1.7(3), N(10)-C(11)-N(12)-C(13) = −4.0(3), N(10)-C(11)-N(12)-C(16) = 155.66(19), N(17)-C(11)-N(12)-C(13) = 177.99(19), N(17)-C(11)-N(12)-C(16) = −22.4(3).

**Figure 4:**
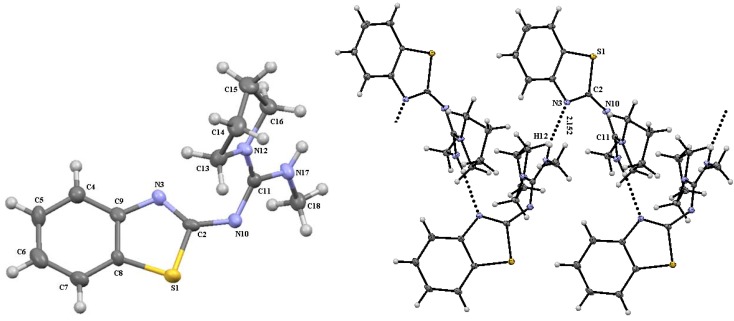
Molecular structure of guanidine **10i** (**left**) and intermolecular interactions (**right**). Selected bond lengths (Å) and angles (°): S(1)-C(2) = 1.779(7), N(3)-C(2) = 1.310(8), N(10)-C(2) = 1.329(9), N(10)-C(11) = 1.332(8), N(12)-C(11) = 1.340(8), N(17)-C(11) = 1.330(7), C(2)-N(10)-C(11) = 122.0(5), S(1)-C(2)-N(3) = 113.4(5), S(1)-C(2)-N(10) = 117.3(5), N(3)-C(2)-N(10) = 129.1(6), S(1)-C(8)-C(9) = 109.9(5), N(3)-C(9)-C(8) = 114.8(5), N(10)-C(11)-N(12) = 123.5(5), C(8)-S(1)-C(2)-N(3) = −1.0(5), C(8)-S(1)-C(2)-N(10) = −176.8(5), S1-C2-N10-C11 = −149.3°(5), C(11)-N(10)-C(2)-N(3) = 35.8(10), N(12)-C(11)-N(10)-C(2) = 39.2(9), N(17)-C(11)-N(10)-C(2) = −144.6(6), N(10)-C(11)-N(12)-C(13) = 13.2(9), N(10)-C(11)-N(12)-C(16) = 177.3(6), N(17)-C(11)-N(12)-C(13) = −163.0(6), N(17)-C(11)-N(12)-C(16) = 1.0(9).

Nonsymmetrical guanidines **10f** and **10i** were crystallized from ethanol; their molecular structures are shown in Figure 3 and Figure 4, respectively. The aniline N-H proton in **10f** is intramolecularly bridged with benzothiazole nitrogen [H17∙∙∙N3 = 2.13 Å, N17∙∙∙N3 = 2.697(3) Å, N17-H17∙∙∙N3 = 123°], forcing the guanidine group to be in the same plane of the benzothiazole ring [N17-C11-N10-C2 = −1.7(3)°] and [N12-C11-N10-C2 = −179.62(17°)]. In addition, two phenyl hydrogen atoms make intermolecular CH∙∙∙π contacts: H19 with guanidine carbon atom [H19∙∙∙C11 = 2.792 Å, C19∙∙∙C11 = 3.684(3) Å, C19-H19∙∙∙C11 = 160.9°; symmetry code: 1−x, −y, 1−z], and H20 with the π electrons of the benzothiazole aromatic ring [H20∙∙∙π = 3.130 Å, C20∙∙∙π = 3.898(3) Å, C20-H20∙∙∙π = 141.2°, symmetry code: 1−x, −1/2+y, 1/2−z]. 

Moreover, in the case of guanidine **10i**, derived from two alkylamines, the conformation is not fixed by intramolecular hydrogen bonding as in **10f**, the methylamine N-H proton is intermolecularly hydrogen bonded with benzothiazole nitrogen of a second molecule and so on to get an helix-like polymer [H17∙∙∙N23 = 2.16Å, N17∙∙∙N23 = 2.973(6), N17-H17∙∙∙N23 =158°; symmetry code: x, y ,z and H37∙∙∙O3 = 2.19 Å, N37∙∙∙O3 = 2.997(7) Å, N37-H37∙∙∙O3 =157°; symmetry code: x, 1−y, z], this forces the plane of the guanidine system approximately 35° out of the benzothiazole mean plane [N17-C11-N10-C2 = −144.6(6)°, N12-C11-N10-C2 = 39.2 (9)°] in agreement with the steric demand of the pyrrolidine moiety.

The proton NMR chemical shift of the NH protons of compounds **10f** and **10i** are in 11.5 and 8.9 ppm respectively. These results show that aniline N-H hydrogen atom of guanidine **10f** is involved in hydrogen bonding in solution whereas the NH of **10i** is not. This result is explained because of, in the case of compound **10f**, the electron pairs of the aniline nitrogen atom are conjugated with the aromatic ring making the NH more acid and thus able for hydrogen bonding. The same effect is observed in the ^1^H-NMR spectra of the series of non-symmetric guanidines based on aniline derivatives. The NH protons are in 11.2 for **10e**, 11.6 for **10f** and 12.4 for **10g**. This intramolecular interaction, make the benzothiazole ring and the guanidine system to be in the same plane, increasing the electronic protection on C2 atom and, shifting the corresponding ^13^C-NMR signals by approximately 3 ppm to lower frequencies, in comparison with guanidines without this group, Table 2.

## 3. Experimental

### 3.1. General Procedures

Melting points were measured on an Electrothermal IA apparatus and are uncorrected. IR spectra were recorded in a film on ZnSe using a Perkin-Elmer 16F PC IR spectrophotometer. ^1^H and ^13^C-NMR spectra were recorded on a Varian Mercury 300 MHz (^1^H, 300.08; ^13^C, 75.46 MHz). The spectra were measured with tetramethylsilane as internal reference following standard techniques. Physicochemical data is listed in Table 3. Crystallographic data (excluding structure factors) for the structures in this paper have been deposited in the Cambridge Crystallographic Data Centre as supplementary publication numbers CCDC **10f** (820770), **10i** (820769), and **9a** (820768). A summary of collection and refinement X-ray data are listed in Table 4. For this compound, H atoms were treated as riding atoms, with C–H distances in the range of 0.93–0.96 Å and N-H distances of 0.82 Å. X-ray diffraction cell refinement and data collection: BRUKER SMART APEX Diffractometer and SAINT [57], programs used to solve structures: SHELXS-97 [58], software used to prepare material for publication: PLATON [59] and *WinGX* [60]. 2-Aminobenzothiazole **4** was a commercial product. Dimethyl benzo[*d*]thiazol-2-ylcarbonodithioimidate **5** was prepared according to a literature procedure [7].

**Table 3:** Complementary data of the starting materials **4**, **5**, **9a** and guanidines **10b**–**j**.

Comp.	Yield (%)	Physical appearance	M.p. (°C)	υ (cm^−1^)	*m/z* (%M^+^)	Elemental analysis Found (calculated)		
						C	H	N
**4**	SM	White solid	126–129					
**5**	82	Yellow powder	72–73	509, 1464	254(20)	47.05(47.24)	3.95(3.94)	11.13(11.02)
**9a**	62	Colorless crystals	198–199	2186, 1600, 1580	175(100)	54.02(54.85)	3.03(2.85)	23.73(24.00)
**10b**	88	White powder	158–160	3406, 3260, 1624	206(100)	52.14(52.42)	4.88(4.85)	27.20(27.18)
**10c**	92	White powder	242–244	3395, 3161, 1609, 1547	246(100)	58.13(58.53)	5.71(5.69)	22.40(22.76)
**10d**	76	White powder	148–150	3436, 3198, 1613, 1568		57.42(62.68)	4.57(4.48)	19.05(20.89)
**10e**	89	White powder	145–147	3418, 3200, 1597, 1560		63.14(63.83)	4.98(4.96)	19.95(19.86)
**10f**	90	Colorless crystals	184–186	3395, 3161, 1609, 1547		66.99(67.08)	5.70(5.59)	17.72(17.39)
**10g**	60	White powder	127–129	3400, 1613, 1580	344(19)	68.19(69.76)	4.72(4.65)	16.17(16.28)
**10h**	90	Brownish liquid		1602, 1574	220(100)	54.80(54.54)	5.49(5.45)	24.24(25.45)
**10i**	92	Colorless crystals	136–137	3210, 3080, 1588, 1524		59.63(60.0)	6.24(6.15)	21.71(21.54)
**10j**	89	Brownish liquid			300(100)	63.2512(64.00)	5.9812(6.66)	19.12(18.66)

**Table 4:** .X-ray crystal data of compounds **10g**, **10i** and **9a**.

Compound	10f	10i	9a
**Unit cell information**			
Cell axes [Å]a	11.3477[13]	14.3400[20]	5.6230[10]
**b**	9.0463[11]	7.8188[12]	8.2300[9]
**c**	16.5004[19]	24.1730[40]	17.2290[10]
Cell angles [deg]α	90.000[0]	90.000[0]	90.000[0]
β	101.858[2]	101.858[2]	90.000[0]
γ	90.000[0]	90.000[0]	90.000[0]
Crystal system	Monoclinic	Orthorhombic	Monoclinic
Space group	P 2_l_/c	P na2_l_	P 2_l_/c
Molecular Formula	C_18_H_18_N_4_S	C_13_H_16_N_4_S	C_8_H_5_N_3_S
Density [g cm^−1^]	1.29	1.28	1.46
Formula weight	322.4	520.7	175.2
No. Form. Units Z	4	4	4
**Reflection data**			
No. Meas.	15334	22566	3406
No. Uniq.	2920	4245	1544
No. Obs.	2630	3049	1278
**Current refinement**			
No. Reflen.	2920	4245	1544
No. Param.	208	325	110
Delta-rho[eÅ^−3^]max, min	0.242, −0.280	0.922, −0.272	0.274, −0.283
R_all, R_obs	0.054, 0.049	0.102, 0.073	0.054, 0.049
wR2_all, wR2_aobs	0.125, 0.121	0.189, 0.166	0.127, 0.116

### 3.2. General Procedure to Get Isothiourea Intermediates *8*

In a 100 mL flask, dimethyl benzo[*d*]thiazol-2-ylcarbonodithioimidate **5** (1.0 g, 3.94 mmol) was dissolved in ethanol (10 mL), three molar equivalents of ammonia, or one molar equivalent of the respective aliphatic or aromatic amine were added and the mixture was stirred for 72 h, in the case of ammonia, 48 h in the case of alkylamine or pyrrolidine and 24 h in refluxing in the case of aniline to get the corresponding isothiourea compounds.

### 3.3. General Procedure to Obtain Guanidines *10*

In a 100 mL flask, isothiourea compound **5** (1.0 g, 3.94 mmol) was dissolved in ethanol (10 mL) with one molar equivalent of the corresponding amine and refluxed for 16 h to get guanidines **10b**–**e**,**g**–**j** or from carboimidate **5** with 2 molar equivalents of the amine in refluxing ethanol for 16 h, in the case of alkylamines, or refluxing DMF, in the case of aniline, to get the corresponding guanidine compounds **10h**,**j**. The solvent was eliminated by evaporation; the resulting solid was washed with cold ethanol, ketone or chloroform, then dissolved in ethanol and after precipitation or crystallization, filtered and air dried to give a white solid.

## 4. Conclusions

We have demonstrated the preparation of symmetric and non-symmetric guanidines from the reaction of dimethyl benzo[*d*]thiazol-2-ylcarbonodithioimidate (**5**) and primary or secondary amines in refluxing ethanol, through the displacement of two molecules of HSMe. The reaction proceeds through isothiourea intermediates which, in strongly basic media, are transformed in (*Z*)-2-cyanamidabenzothiazoles. Alkylamines are nucleophilic enough to easily perform both substitutions leading to guanidines, whereas the second substitution with aniline requires harsh conditions. Intramolecular hydrogen bonding between the NH of the aniline group and benzothiazole nitrogen in *S*-methylisothiourea **8d**, leads to a *cis*-disposition between them and thus controlling the stereochemistry of the second substitution. The NH of alkylamines is not acidic enough to form intramolecular hydrogen bonding and intermolecular interactions are found instead. In any case the preferred rotamer observed in the solid state is *trans* to the sulfur atom of benzothiazole ring.
